# Two case reports of skin vasculitis following the COVID-19 immunization

**DOI:** 10.1515/med-2022-0608

**Published:** 2022-12-09

**Authors:** Anželika Chomičienė, Kęstutis Černiauskas, Kotryna Linauskienė, Raimundas Meškauskas, Laura Malinauskienė

**Affiliations:** Vilnius University, Medical Faculty, Institute of Clinical Medicine, Clinic of Chest Diseases, Immunology and Allergology, Department of Pulmonology and Allergology, Vilnius, Lithuania; Pathology Department, National Center of Pathology, Affiliate of Vilnius University Hospital Santaros Klinikos, Vilnius, Lithuania; Vilnius University Hospital Santaros Klinikos, Department of Pulmonology and Allergology, Santariškių Str. 2, Vilnius LT-08661, Lithuania

**Keywords:** case report, COVID-19 disease, vaccine, vasculitis, plasmapheresis, mRNA vaccine, adverse events

## Abstract

The coronavirus 2019 (COVID-19) disease is now responsible for one of the most challenging and concerning pandemics. Since December 2020, the world has had access to COVID-19 prophylaxis; thus, we encounter adverse events from vaccination more often due to the vast vaccination range. We present two case reports of difficult-to-treat skin vasculitis due to COVID-19 vaccination that were successfully treated in a tertiary-level university hospital. When encountering systemic treatment, resistant skin vasculitis plasmapheresis could be a choice of treatment.

## 1 Introduction

The coronavirus 2019 (COVID-19) pandemic, caused by the severe acute respiratory syndrome coronavirus 2 (SARS-CoV-2), has had terrible health, economic, and social consequences. As vaccines for the novel COVID-19 disease are already authorized in many countries around the world, knowledge about adverse events following immunization (AEFI) is essential for medical practitioners and health authorities in the context of the safety and tolerability of vaccines. An AEFI is defined as any untoward medical occurrence that follows immunization and does not necessarily have a causal relationship with the use of a vaccine [1]. We present two cases of vasculitis after immunization with the Pfizer Comirnaty COVID-19 vaccine.

## 2 First case

**Figure 1:**
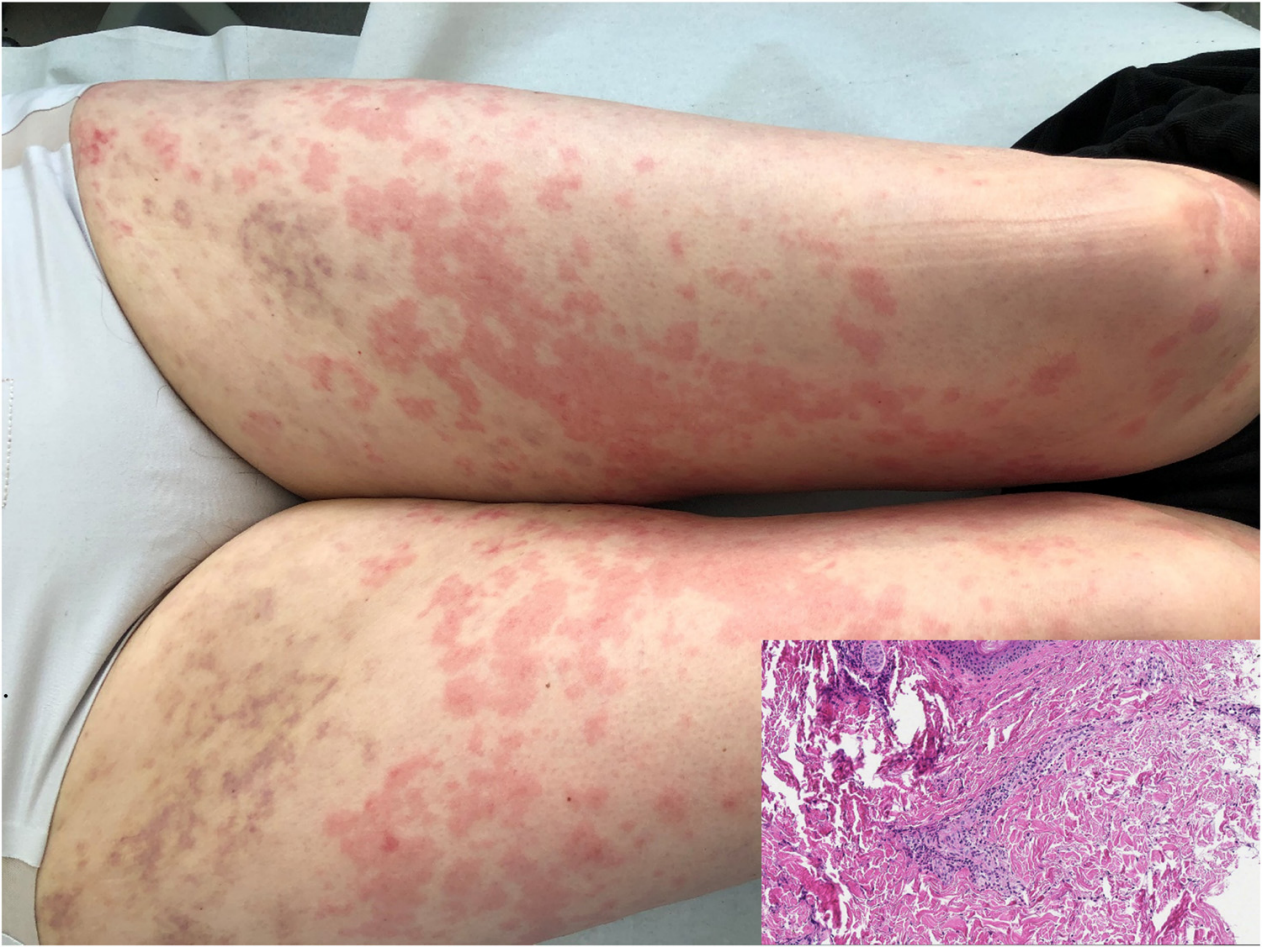
Urticarial and palpable non-blanching purpuric rash compatible with mild mixed perivascular infiltration of lymphocytes and neutrophils in the superficial dermis, oedema of blood vessels endothelium, no vessels wall necrosis identified.

A 65-year-old otherwise healthy woman was consulted at our center in February 2021 because of severe urticaria and angioedema. She reported that hives appeared 12 days post-vaccination with the second dose of the Pfizer Comirnaty COVID-19 vaccine and lasted for 2 weeks. She denied having other symptoms such as fever, fatigue, headache, nausea, muscle or joint pains, a local reaction at the injection site, or swelling in the underarm. Urticarial rash and angioedema episodes of the lips and ears were associated with burning sensation and pruritus. On the initial physical examination, next to typical urticaria on the limbs and trunk, some palpable non-blanching purpuric papules were noticed on the thighs (Figure 1). The rest of the physical examination was unremarkable. The patient did not refer to any relevant past medical history. She did not take any medications and had no allergies to vaccines, drugs, or food in the past. Full blood workup, including erythrocyte sedimentation rate (ESR), C-reactive protein (CRP), and immunologic tests (C3, C4, antinuclear antibodies (ANA), antineutrophil cytoplasmic antibody (ANCA), and cryoglobulins), was done the following day, and all were normal, except for a moderately elevated white blood cells (11.9 × 10^9^/L) with predominant neutrophils (72.2%), which was attributed to previous treatment with corticosteroids. The Chest X-ray showed no abnormalities. There were several erythrocytes in the urine analysis. A skin biopsy was taken. Histopathologic findings revealed mild mixed perivascular infiltration of lymphocytes and neutrophils in the superficial dermis, a thin interstitial neutrophil infiltrate of the derma, oedema of the vascular endothelium, and vessel wall necrosis, but no signs of IgA vasculitis were found (Figure 1).

The urticarial vasculitis (UV) was diagnosed, and the patient was treated with 32 mg of oral methylprednisolone, 20 mg omeprazole, and 40 mg per day rupatadine. She also received several intravenous injections of dexamethasone. Plasmapheresis is often used in our clinic in cases when other therapies fail. Plasmapheresis was started after 6 days of unsuccessful treatment with medications, and the patient had an overall five procedures. After two procedures of plasmapheresis, the urticaria disappeared and did not reoccur. Therefore, systemic steroids were tapered gradually for 1 month. The patient recovered completely. She was followed up for 1 year.

## 3 Second case

**Figure 2:**
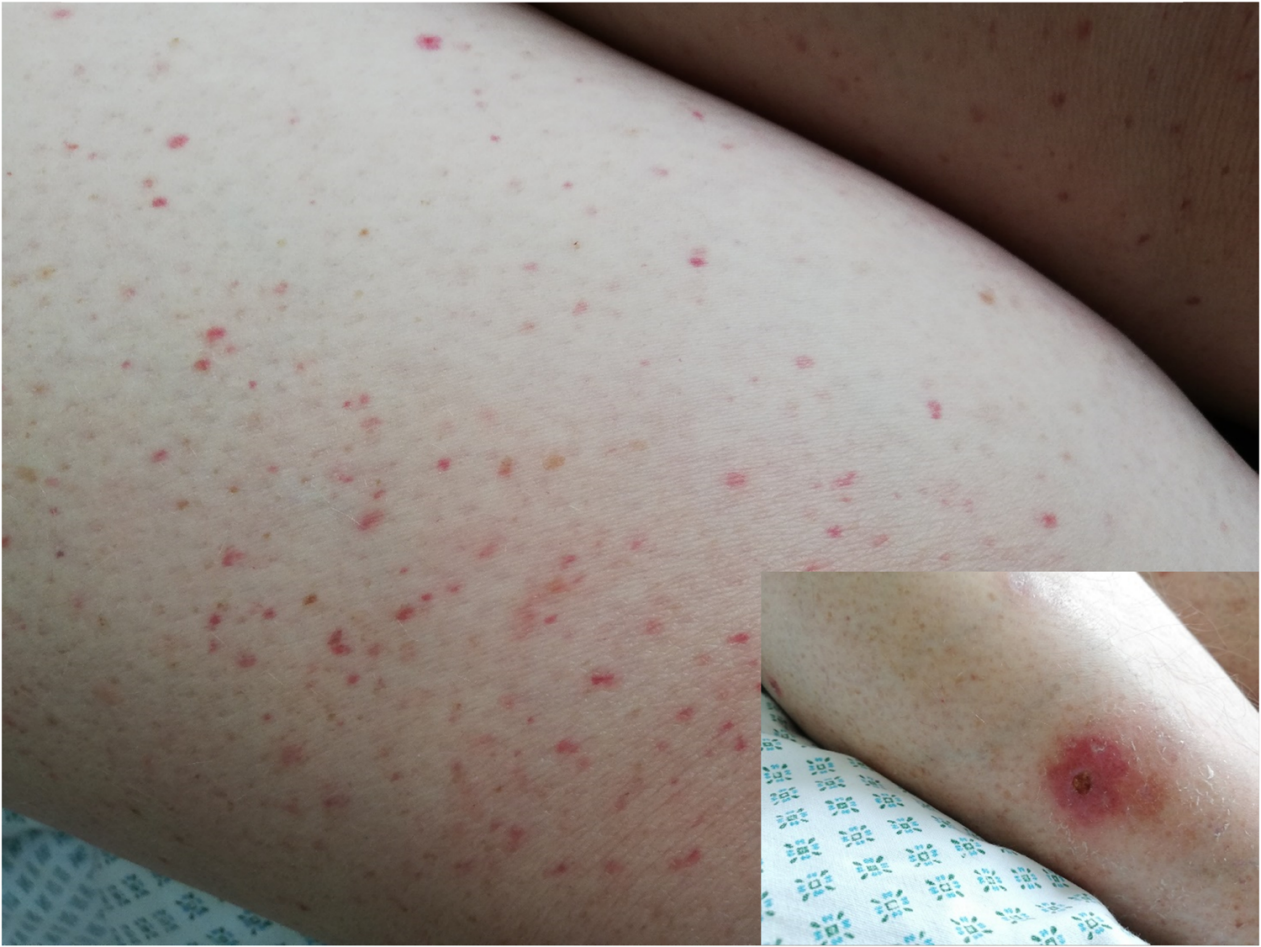
A palpable non-blanching purpuric rash on the right thigh and a sore with necrosis signs on the right shin.

**Figure 3:**
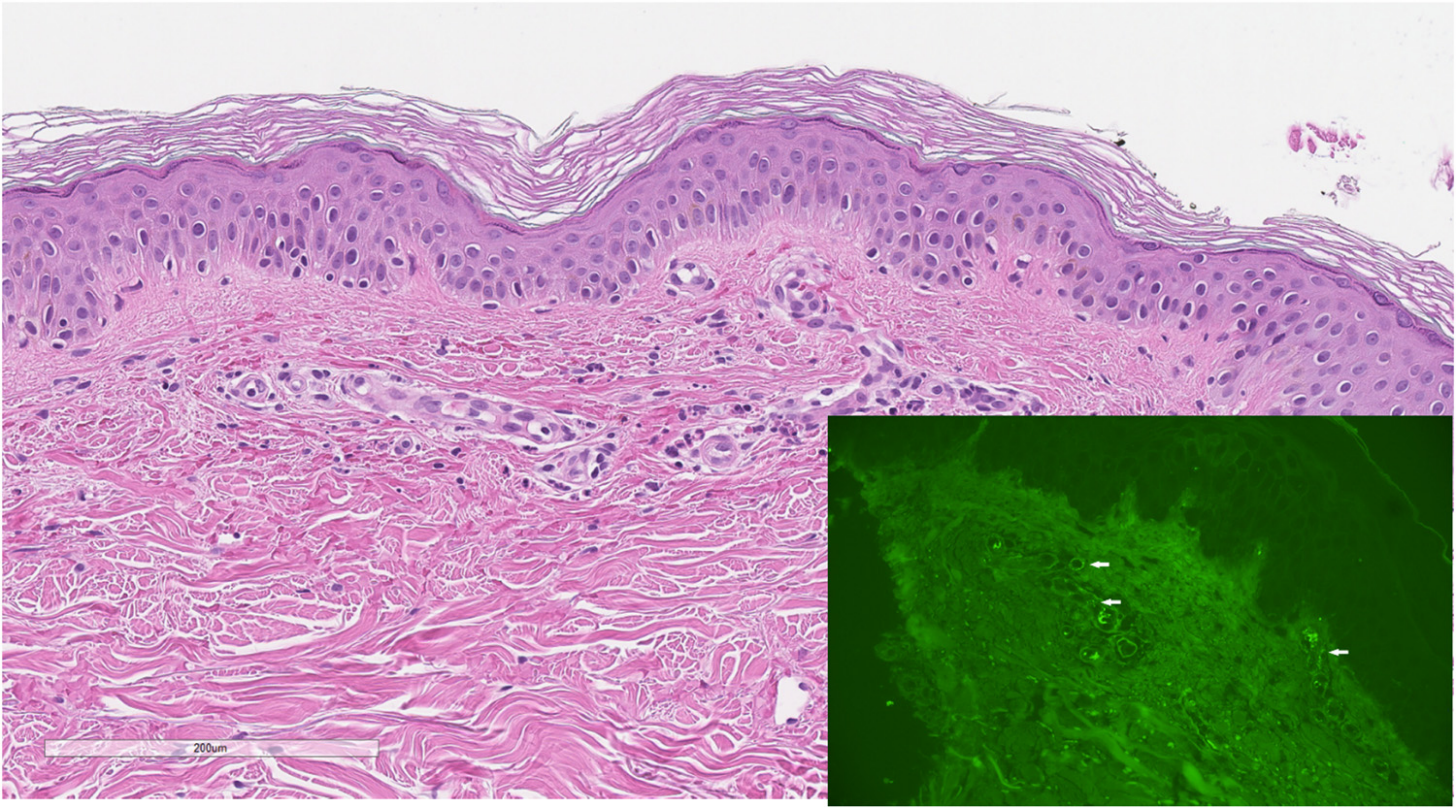
Small vessels oedema of endothelium with perivascular leukocytoclasis, erythrocytes extravasation with focal IgA deposition in the vessels.

A 49-year-old woman was consulted by an allergologist and clinical immunologist because of a rash on her extremities. The rash appeared 3 weeks after the first dose of the Pfizer Comirnaty COVID-19 vaccine. The patient felt weakness and pain in the shins. Earlier the patient was consulted by another allergologist, vasculitis was suspected, and treatment with triamcinolone acetonide 40 mg injection started every 3 weeks. The improvement was only temporary, and after 4 months, she was consulted by the rheumatologist and hospitalized at the Center for Rheumatology with suspicion of systemic vasculitis. On physical examination, a palpable non-blanching purpuric rash on all extremities (Figure 2) was evident with some small sores with necrosis on the right shin (Figure 2). No other symptoms (fever, fatigue, or joint pain) were observed. The patient had leukocytosis (up to 12.57 × 10 9/L) with 74.9% neutrophils and normal platelet (242 × 10^9^/L) and eosinophil (0.00 × 10^9^/L) counts. CRP, ESR, urinalysis, creatinine, liver function tests, C3, and C4 were within normal limits. ANA, ANCA, human immunodeficiency virus, hepatitis B virus surface antigen, and hepatitis C virus antibodies were negative. The chest X-ray showed no abnormalities. Abdominal echoscopy showed hepatosteatosis and calcinate in the right kidney. Histopathologic investigation of the skin biopsy revealed small-vessel oedema of the endothelium with perivascular leukocytoclasis, erythrocytes extravasation, and focal IgA deposition in the vessels (Figure 3). IgA vasculitis (Henoch-Schönlein purpura) was diagnosed. The patient was treated with 32 mg of oral methylprednisolone and a plasmapheresis course. The rash disappeared after 4 weeks. Systemic steroids were tapered gradually for 6 weeks. The patient recovered completely and was followed up for 1 year.

Both of the patients gave written consent for the publication of the case report.

## 4 Discussion

COVID-19 patients present with many extra-respiratory manifestations in addition to respiratory symptoms and fever [2]. A constituent number of case reports and clinical series have already been published describing a complex spectrum of skin manifestations associated with the SARS-CoV-2 infection [3,4,5]. Skin lesions with features of vasculitis have been reported in COVID-19 cases, ranging from mild or asymptomatic to fulminant disease [6,7,8]. In addition, there are some reports in the recent literature of cutaneous vasculitis secondary to COVID-19 vaccination [9,10,11].

Based on the clinical presentation and histological features, a clinical diagnosis of UV was made in our first patient. UV is a small-vessel vasculitis with predominantly skin involvement manifesting as urticarial lesions. Angioedema is present in less than half of patients. UV is attributed to the deposition of immune complexes on the blood vessel wall [11]. UV is more often idiopathic, but in some cases, it could occur secondary to an underlying infection, connective tissue disorders, malignancy, and medications.

IgA vasculitis, or Henoch-Schönlein purpura, is a rare autoimmune disease that usually presents in the paediatric and teenage population. IgA vasculitis is often linked to viral or bacterial infection, and a particular association with vaccination has also been documented. IgA vasculitis is often associated with influenza vaccination and usually occurs within 21 days after administration of the influenza vaccine and most affected patients (73%) are younger than 17 years old, it is usually a self-limiting illness [12]. We present an atypical case report of Ig A vasculitis in a middle-aged woman with a progression of skin rash over 4 months despite treatment with systemic corticosteroids.

Vasculitides have been reported as AEFI with several types of vaccines, and the literature review showed numerous reports of vasculitides following vaccination described in a limited number of controlled studies [13]. An important criterion guiding the causality assessment of each event is the temporal relationship between the vaccine and the event, which is deemed to be in the range of 1–6 weeks for drug and vaccine-induced vasculitis. In our cases, it was 12 and 21 days. According to the descriptive analyses across three international databases, vasculitides were more frequently reported in association with influenza vaccines than with any other vaccine. The distribution of vasculitis events by gender showed a slight female predominance (54.3%). Vasculitis appears to be less frequently reported in the elderly (in 15.5% of vaccinated persons on average). Among those patients with the recorded outcomes, 44.3% recovered, 1% had a fatal outcome, and 55% had not recovered/not resolved or the outcome was unknown [14,15].

The exact pathogenesis of vaccination-associated vasculitis has yet to be determined, but the SARS-CoV-2 vaccine might result in the activation of autoreactive B/T cells, antibody formation, and immune complex deposition within small vessels, leading to activation of the complement system and recruitment of leukocytes. The responsible particle as an antigen for such reactions is unknown; however, inflammatory response to a vaccine component encoding SARS‐CoV‐2 spike glycoprotein, targeting the endothelium and resulting in small-vessel vasculitis, could be hypothesized [11]. We described two cases of different types of skin vasculitis following COVID-19 immunization, successfully treated with plasmapheresis and systemic corticosteroids.

The safety of future vaccination in patients with a prior diagnosis of vasculitis due to a vaccine is debatable.

## Abbreviations

AEFI: adverse events following immunization.
UV: urticarial vasculitis
